# Insights into the pathogenesis of rheumatic and immune diseases from single-cell omics

**DOI:** 10.3389/fimmu.2026.1768719

**Published:** 2026-03-12

**Authors:** Wei Wan, Shiyang Zhang, Yingjie Jiang, Dongbao Zhao

**Affiliations:** 1 Department of Rheumatology and Immunology, The First Affiliated Hospital of Naval Medical University, Shanghai, China; 2 The School of Basic Medical Sciences, Naval Medical University, Shanghai, China; 3 Department of Pathology, The First Affiliated Hospital of Naval Medical University, Shanghai, China

**Keywords:** AS, IgG4-RD, PSS, RA, SLE, SSc, clinical translation, single-cell omics

## Abstract

High-resolution, high-throughput single-cell omics has transformed our understanding of autoimmune disease pathogenesis. We synthesise recent single-cell omics advances across six autoimmune diseases—systemic sclerosis, systemic lupus erythematosus, Sjögren’s syndrome, ankylosing spondylitis, IgG4-related disease and rheumatoid arthritis. We further summarise translational progress in targeted therapies. We delineate core pathological networks shared across these conditions, highlight convergent mechanisms, and provide a mechanistic rationale for the clinical activity of agents such as tofacitinib and abatacept across multiple autoimmune settings. These insights support the feasibility of mechanism-informed, cross-disease targeting—deploying shared pathway interventions across distinct clinical entities. Finally, we discuss current technical and interpretative challenges and outline future directions for mechanistic discovery, target prioritisation and precision medicine.

## Graphical Abstract

**Figure:**
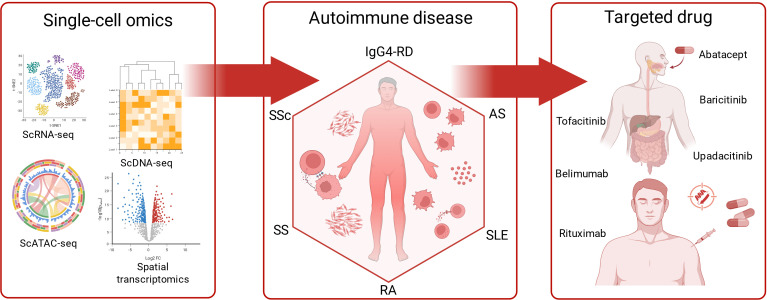

## 1 Introduction

Single-cell omics comprises a suite of technologies that interrogate individual cells as the fundamental unit of analysis. By profiling gene expression, genetic variation and epigenetic regulation at cellular resolution, these approaches enable precise dissection of cellular functions, differentiation trajectories and disease mechanisms. A major strength of integrative single-cell multi-omics lies in combining complementary layers of information, including transcriptomes, immune repertoires, spatial context and epitope measurements. Such integration supports mechanistic inference, sharpens interpretation of cellular heterogeneity, and delineates the spatial architecture, intercellular communication and microenvironmental regulation that shape tissue states (1, 2). Autoimmune diseases arise when immune tolerance fails and immune effector pathways are misdirected against self tissues. Accumulating evidence implicates a convergence of genetic susceptibility, environmental triggers and aberrant activation of B and T cells, culminating in tissue injury; however, the initiating events and causal hierarchy remain incompletely defined. Collectively, autoimmune diseases impose a substantial and multifaceted burden on patients, health-care systems and society (3, 4). Accordingly, current efforts focus on resolving disease mechanisms and prioritising actionable targ*ETS* to accelerate clinical translation and inform effective therapeutic strategies. Single-cell omics has become indispensable for mapping the cellular ecosystems of autoimmune tissues and their microenvironmental cues, thereby providing a mechanistic framework for understanding pathogenesis and guiding target discovery.

This review leverages single-cell omics evidence to delineate pathogenic networks shared across autoimmune diseases and to connect these networks with translatable therapeutic targ*ETS*. Focusing on systemic sclerosis, systemic lupus erythematosus, Sjögren’s syndrome, ankylosing spondylitis, IgG4-related disease and rheumatoid arthritis, we compare reproducible pathogenic cell states and intercellular communication features across tissues and cohorts, distilling five recurrent modules: IFN-I signalling, aberrant T–B cell crosstalk, myeloid inflammatory amplification networks, fibroblast-driven immunopathological remodelling, and pathways of programmed cell death. Within this integrative framework, we synthesise clinical evidence supporting candidate targ*ETS* and discuss priorities for future investigation.

## 2 Methods

**Figure 1:**
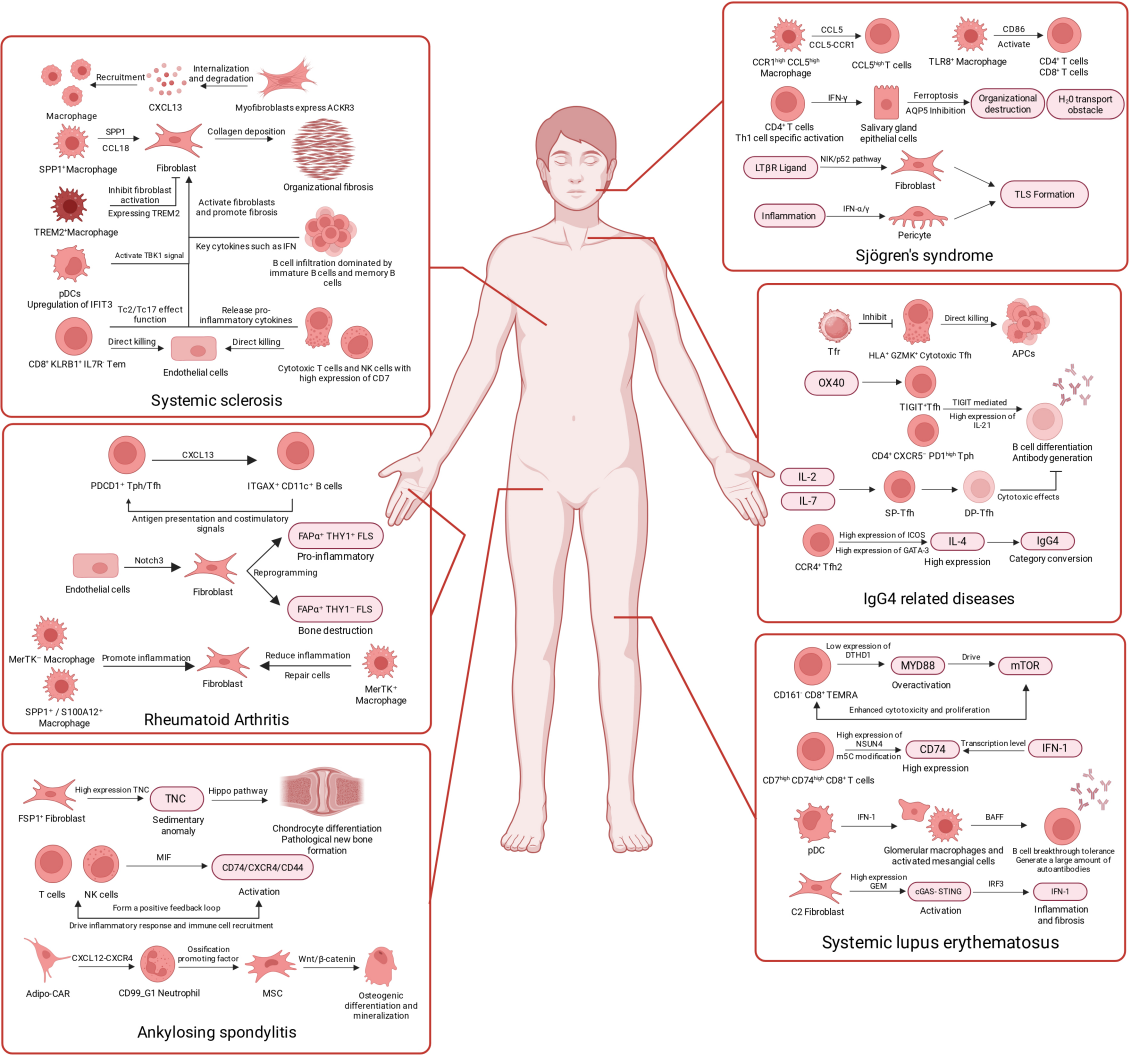
The latest single-cell omics advances in systemic sclerosis, ankylosing spondylitis, Sjogren’s syndrome, IgG4-related diseases, and systemic lupus erythematosus. Created by BioRender.

We conducted computer-assisted searches of PubMed, Web of Science, Embase and CNKI. The search window spanned January 2019 to December 2025, with emphasis on high-quality studies from the past three years to ensure contemporaneity. Search terms covered technologies (e.g., single-cell omics, single-cell RNA sequencing, single-cell multi-omics and spatial transcriptomics), diseases (e.g., autoimmune disease, systemic sclerosis, systemic lupus erythematosus, Sjögren’s syndrome, ankylosing spondylitis, IgG4-related disease and rheumatoid arthritis) and therapeutics (e.g., targeted therapy, clinical translation and pathogenesis). We included original studies interrogating the pathogenesis of these six diseases at single-cell resolution, studies reporting novel target discovery and clinical evaluation of targeted agents, and high-quality peer-reviewed reviews or meta-analyses. We excluded studies unrelated to the six core diseases, case reports with very small sample sizes or lacking key single-cell data, duplicate publications, and articles for which full texts were unavailable. Two investigators independently screened the literature. During the initial screening, titles and abstracts were assessed to remove clearly irrelevant records. In full-text review, we extracted core pathogenic cell populations, key signalling pathways and the latest progress in clinical drug trials for each disease. We then systematically categorised and synthesised included studies to identify shared pathological networks across the six diseases—encompassing IFN-I signalling, aberrant T/B cell interactions, a macrophage-centred MIF axis, fibroblast-mediated immune remodelling and programmed cell death, and used this framework to assess the theoretical plausibility of cross-disease targeting. For each disease section, we summarise key pathogenic cell subs*ETS*, representative interaction axes and translational leads; in the Discussion, we integrate the five shared mechanisms from a cross-disease perspective to build a coherent evidence-to-target narrative. To facilitate rapid appreciation of the principal cell subs*ETS*, interaction axes and pathway frameworks revealed by single-cell omics, we summarise the core mechanistic points for each disease in **Figure 1**.

### 2.1 Systemic sclerosis

Systemic sclerosis is a prototypical fibrotic autoimmune disease; here we focus on how vascular injury and fibroblast activation couple with immune-cell infiltration, providing a conceptual foundation for the cross-disease prominence of fibroblasts and IFN-I discussed below. At cellular resolution, systemic sclerosis is characterised by heightened apoptosis-associated programmes in arterial endothelial cells, accompanied by expansion of tip and proliferative endothelial subs*ETS* and increased expression of *ETS* family transcription factors (5). Within fibrotic skin, endothelial cells upregulate *ACKR1*, which is linked to leukocyte infiltration and fibrotic remodelling (6). Notably, a subset of endothelial cells displays robust IFN-I signalling together with fibroblast-like features, implicating IFN-I–associated endothelial-to-mesenchymal transition (EndoMT) in fibrogenesis (7). Importantly, vasculopathy does not occur in isolation but emerges from dynamic crosstalk among endothelial cells, pericytes and infiltrating immune populations. Macrophages exert context-dependent, bidirectional effects across disease stages. Integrative spatial and single-cell analyses indicate that fibroblast–macrophage crosstalk drives fibrotic expansion and tracks closely with clinical progression (8). *ACKR3* is selectively expressed in myofibroblast precursors and may tune *CXCL12*–*CXCR4* signalling to recruit pro-inflammatory macrophages. *SPP1*^+^ macrophages represent a major pro-fibrotic subset in SSc-ILD, marked by high expression of fibrogenic mediators such as *SPP1* and *CCL18 (*9). Beyond promoting fibrosis, macrophages can also engage protective programmes. *TREM2*^+^ macrophages can restrain skin fibrosis in a *TREM2*-dependent manner, supported by functional validation and single-cell profiling (10). Among macrophage precursors, *CD16*^+^*FCGR3A*^+^ non-classical monocytes and *IRF7*^+^*STAT1*^+^ inflammatory monocytes exhibit pronounced IFN responses, including high *ISG15* expression (11). Single-cell datas*ETS* further suggest a reduction of M2-like circulating monocytes in blood alongside accumulation of M2-polarised macrophages in affected tissues, consistent with compartmental trafficking into lesions. Other immune compartments—including dendritic cells, T cells, B cells and NK cells—also undergo substantial state shifts, collectively shaping disease progression. pDCs promote fibroblast proliferation, migration and contractility, and induce fibrotic protein expression via the *IFIT3*–*TBK1* axis (12). Within the T-cell compartment, *CD8*^+^*KLRB1*^+^*IL7R*^-^ effector-memory cells show enhanced cytotoxicity and Tc2/Tc17 programmes, which may contribute to tissue injury and fibrosis (13). A second subset comprises *CD8*^+^*KLRG1*^+^*IL7R*^-^ T cells with depletion/exhaustion features and long-lived effector-like transcriptional signatures, potentially sustaining chronic inflammation through persistence. Infiltrating B cells—predominantly naïve and memory subs*ETS*—can elicit pro-inflammatory and pro-fibrotic responses in fibroblasts, partly through cytokines such as *TNF (*14). NK cells and cytotoxic T lymphocytes infiltrate skin and show high *CD7* expression; through reinforced cytotoxic and inflammatory programmes and reciprocal interactions with fibroblasts, they may exacerbate cutaneous inflammation, injury and fibrosis (15). Single-cell evidence in systemic sclerosis links endothelial stress, myeloid inflammatory activation and fibroblast-lineage remodelling into a mutually reinforcing network, underscoring IFN-I signalling and fibroblast state transitions as central nodes for cross-disease integration.

### 2.2 Systemic lupus erythematosus

Systemic lupus erythematosus is characterised by IFN-I–driven systemic inflammation and multi-organ injury; here we examine IFN-I-reinforced myeloid and lymphoid states and how key communication circuits sustain autoreactive B cells and amplify inflammatory responses. Type I interferon (IFN-I) signalling is central to SLE pathogenesis (16). In SLE, IFN-I programmes are prominent in PBMC T cells, renal *CD8*^+^ T cells and NK cells, and epidermal keratinocytes (17). Across these compartments, interferon-stimulated genes (ISGs)—including *ISG15*, *IFI44L*, *IFI6* and *IFI27*—are consistently upregulated in keratinocytes and T cells. B cells also show augmented IFN-I signalling and features consistent with senescence, with increased expression of *ISG15*, *IFI44L* and *IFITM1 (*18). Across immune lineages in SLE, heightened IFN signalling and its downstream mediators represent a pervasive, shared signature. Classical monocytes—often exhibiting the strongest ISG signature—are expanded in SLE and display functional perturbations (19, 20). Upregulation of mononuclear-cell transcripts—including *IFI6*, *IFIH1*, *SCO2*, IRF family members, *IFITM3*, *S100A9*, *CCL4*, *CXCL8*, *ISG15*, *STAT1* and *IFI44L*—further supports a central role for these cells in inflammatory amplification (21–23). Among these candidates, *IRF1* shows particularly elevated expression and may represent a promising biomarker and therapeutic entry point. At the level of cell–cell communication, monocytes engage broadly with B and T cells through pathways that include *TNF*, *BAFF*, MIF, GALECTIN and ANNEXIN signalling. In T cells, upregulation of *IRF3*, *IRF7* and *STAT1* is consistent with reinforcement of IFN-I responses (24). Single-cell analyses have revealed marked expansion of *CD161*^-^*CD8*^+^ TEMRA cells in SLE (25). Low *DTHD1* expression in this subset suggests excessive activation of the *MYD88* pathway when *DTHD1* is reduced. This in turn augments *mTOR* signalling, promoting proliferation and cytotoxicity, fostering a systemic inflammatory milieu, and reinforcing IFN-I programmes. A previously underappreciated *CD7*^high^*CD74*^high^*CD8*^+^ T-cell subset shows prominent exhaustion signatures and is expanded in SLE (26). Integrative analyses suggest that *NSUN4* upregulation increases *CD74* protein abundance via m^5^C modification, providing a potential post-transcriptional contribution to *CD8*^+^ T-cell exhaustion. Together, these findings position *CD74* as an IFN-I-responsive gene whose transcriptional induction is reinforced by IFN-I signalling and whose sustained expression may be stabilised through an *NSUN4*–m^5^C–dependent post-transcriptional mechanism. Multiple studies nominate *BAFF* as a therapeutically actionable node. IFN-I produced by pDCs and other sources can induce *BAFF* expression, supporting persistence of autoreactive B cells and sustained antibody production. In the kidney, *BAFF* is locally expressed, with glomerular macrophages and activated mesangial cells proposed as major sources (17, 27). Beyond immune lineages, a C2 fibroblast subset has been implicated in SLE. This subset exhibits elevated *GEM* expression and an activated programme that may promote tissue inflammation and fibrotic progression in SLE (28). Activated fibroblasts may also contribute to inflammatory reinforcement by promoting IFN-I production. In SLE, IFN-I signalling spans myeloid and lymphoid compartments to form sustained communication networks and, through key humoral axes, maintains autoreactive B cells—thereby providing a mechanistic rationale for cross-disease targeting of IFN-I and T–B cell crosstalk.

### 2.3 Sjögren’s syndrome

Sjögren’s syndrome is driven by chronic exocrine-gland inflammation and remodelling of intratissue immune architecture; here we highlight macrophage-mediated antigen presentation and chemotactic networks, and the pivotal role of T–B interactions in sustaining inflammation. In pSS, the dominant immune populations include T cells, B cells, NK cells, dendritic cells and monocyte–macrophage lineages, with monocyte–macrophages typically most abundant (29). Across monocyte subs*ETS* in pSS, *TNFSF10* is broadly upregulated, together with enrichment of interferon-related programmes and neutrophil-activation pathways (30). In pSS plasma, elevated *IL-1*5 can induce *CXCR6* and *GZMK* expression in *CD8*^+^ T cells, promoting differentiation towards Trm precursors (31). Within salivary glands, two *CD8*^+^ Trm subs*ETS* are evident: *CD103*^+^ and *CD103*^-^ populations (32). *CD103*^-^*CD8*^+^ Trm cells show higher *GZMK*, *IFN-γ* and HLA-DR expression, consistent with an activated, more cytotoxic state. They constitute a major local source of inflammatory cytokines within the gland. Recent studies collectively map extensive macrophage–T-cell interactions with both *CD4*^+^ and *CD8*^+^ subs*ETS*, offering mechanistic insight into tissue injury. Given their abundance in both PBMCs and glandular tissues, macrophages serve as key intermediaries linking innate and adaptive immunity in pSS (29). *CCR1*^high^*CCL5*^high^ macrophages infiltrating salivary glands secrete *CCL5*, recruiting *CCL5*^high^ T cells via *CCL5*–*CCR1* signalling (33). *TLR8*^+^ macrophages can upregulate *CD86*, thereby priming and activating both *CD4*^+^ and *CD8*^+^ T cells. Under strong interferon signalling, *CD4*^+^ T cells display Th1-skewed activation and produce *IFN-γ*, which has been linked to ferroptosis and suppression of *AQP5* in salivary gland epithelial cells, thereby contributing to tissue destruction and impaired water transport (34, 35). Cytotoxic-gene–high *CD4*^+^ CTLs and *TRAV13-2*^+^*CD4*^+^ T cells show marked expansion in pSS (36). *CD4*^+^ CTLs express *CX3CR1*, *NKG2D*, T-bet and *RUNX3*, alongside reduced or absent *CD28*. These features support efficient recruitment to inflamed tissues, persistence, and direct cytotoxicity. *CD8*^+^ T cells also show substantial clonal expansion with heightened cytotoxicity and high expression of *IFN-γ*, *TNFSF9* and related mediators, likely contributing directly to tissue injury (31, 32). TLS formation is associated with establishment of a pro-inflammatory microenvironment and broader immune activation in pSS. This involves differentiation of *ACKR3*^+^ fibroblasts via *LTβR*-dependent non-canonical *NF-κB* signalling and activation of *CCL21*^+^*CCL19*^+^ pericytes through *TNF*-α signalling (37). *CCL19*^+^*TNFSF13B*^+^ immunofibroblasts engage antigen presentation and *IFN-γ* signalling programmes and appear to form a structural and functional core of TLS. In Sjögren’s syndrome, tissue-specific inflammation and immune-architectural remodelling are mutually reinforcing; macrophage-mediated antigen presentation and chemotaxis bridge innate and adaptive immunity, further underscoring the cross-disease generality of T–B interactions.

### 2.4 Ankylosing spondylitis

Ankylosing spondylitis is characterised by enthesitis and structural damage; here we emphasise myeloid inflammatory amplification loops and fibroblast-driven tissue remodelling signals that couple inflammation to matrix reconstruction. At the molecular level, single-cell studies nominate *AIF-1* as a potential biomarker and *XBP1* as a candidate therapeutic target (38, 39). In NK cells, granzyme transcripts (*GZMA*, *GZMB* and *GZMM*) are reduced, whereas *IL2RB*, *CD247*, *PLEKHF1*, *S1PR5* and *FGFBP2* are increased relative to other lineages (40, 41). *CD74* is markedly upregulated in T cells and NK cells in AS (42). XIST and *MNDA* are highly expressed across multiple monocyte subs*ETS* (43). Transcriptomic changes increasingly suggest a progression framework characterised by cellular stress, epigenetic reprogramming and immune dysfunction in AS. Endoplasmic reticulum stress and the unfolded protein response, altered chromatin accessibility at immune loci, and recruitment yet functional impairment of NK cells may together shape the inflammatory milieu. At cellular resolution, *CD8*^+^ T cells show aberrant *NF-κB* activation with increased AP-1 transcription factor activity (44). This may promote persistent, self-sustaining *TNF* signalling and thereby contribute to chronic inflammation. In the joint cavity, *CD8*^+^ cytotoxic T cells upregulate inhibitory receptors (*PD-1*, *TIGIT* and LAG-3) while downregulating *CD127 (*45). Despite this phenotype, they retain substantial effector capacity rather than displaying overt functional exhaustion. Neutrophils are abundant in AS tissues and show high CAT expression (46, 47). Elevated CAT may mitigate oxidative stress, supporting prolonged neutrophil survival. Surviving neutrophils can then express high levels of MIF, amplifying inflammatory signalling and potentially creating a self-reinforcing loop. OAt the interaction level, inflammatory cues in AS induce FSP1^+^ fibroblasts to upregulate *TNC*, promoting extracellular matrix *TNC* deposition (48). This may promote chondrogenic differentiation and pathological new bone formation through Hippo pathway signalling. T cells and NK cells may dominate intercellular communication via paracrine and autocrine MIF–*CD74*/*CXCR4*/*CD44* signalling, driving inflammation and immune recruitment (42). A high proportion of double-TCR T cells has also been reported in AS, potentially enhancing self-antigen recognition, evading thymic negative selection, shaping Th17/Treg polarisation and facilitating interconversion between T-cell states (49). At inflamed spinal entheses, Adipo-CAR cells recruit CD99_G1 neutrophils via the *CXCL12*–*CXCR4* axis. These CD99_G1 neutrophils then express and secrete pro-ossification mediators targeting MSCs and osteogenic-lineage cells, activating pathways such as Wnt/β-catenin (46). This cascade may drive osteogenic differentiation and mineralisation, culminating in pathological new bone formation. Single-cell atlases of AS indicate parallel activation of myeloid inflammatory amplification and fibroblast-mediated matrix remodelling, offering a cellular-state explanation for inflammation-driven structural damage.

### 2.5 IgG4-related disease

IgG4-related disease is characterised by inflammatory fibrosis and plasmacytic infiltration; here we focus on T-cell–dependent B-cell responses and the regulatory circuits through which aberrant humoral immunity couples to fibrotic progression. TOP2A^+^ T cells express stem-like markers and may differentiate towards Tfh states (50). *ICOS*^+^*PD-1*^+^ B cells display Tfh-like features and may represent an intermediate state en route to IgG4^+^ plasma cells. IgG4-RD lesions are enriched for *CD4*^+^ cytotoxic T cells expressing *GZMA*, *GZMK* and *SLAMF7*, proposed as key drivers of inflammatory fibrosis (51). In peripheral blood, *CD4*^+^ CTLs and *GNLY*^+^*CD8*^+^ CTLs are increased and exhibit high cytotoxic and chemotactic programmes (9). CTLs show high *RUNX3*, *TBX21* and *EOMES* expression, correlating with cytotoxicity. *CD8*^+^ central memory T cells and *TIGIT*^+^*CD8*^+^ cytotoxic T cells are also expanded (52). These shifts suggest a maladaptive cycle in which persistent antigenic stimulation repeatedly mobilises effector responses and strengthens cytotoxic and chemotactic capacity. Yet incomplete antigen clearance may culminate in progressive dysfunction and exhaustion under chronic stimulation. B cells have been proposed to function as antigen-presenting cells that activate and sustain *GZMK*^+^ CTLs (53). Activated cytotoxic populations may contribute to fibrosis by producing mediators such as *AREG* and *TGF-β*, thereby promoting tissue injury and remodelling. At the level of cell–cell communication, multiple studies converge on an “accelerator–brake” architecture in IgG4-RD pathogenesis. Within TLO structures, a distinct HLA^+^*GZMK*^+^ cytotoxic Tfh subset has been described (54). These cells may directly target MHC-II–expressing cells, thereby contributing to tissue injury and fibrotic progression. Enrichment of Tfr cells may counterbalance this response by restraining cytotoxic Tfh activity. In retroperitoneal and salivary gland tissues, *CD4*^+^*CXCR5*^-^*PD-1*^high^ Tph cells are markedly increased (55). Tph cells can regulate *IL-21* via *TIGIT* and thereby promote B-cell differentiation and antibody production. *OX40* co-stimulatory signalling is aberrantly activated in IgG4-RD. This may act on *TIGIT*^+^ Tfh cells to increase *IL-21*, thereby driving B-cell–mediated humoral responses (56). *TIGIT* has therefore been proposed as a marker of disease activity. In affected tissues such as submandibular gland, *IL-2* and *IL-7* can synergistically stimulate SP-Tfh cells and promote conversion to DP-Tfh cells (57). DP-Tfh cells may then exert cytotoxic restraint on memory-B-cell differentiation into antibody-secreting cells, providing negative feedback on IgG4 production. *CCR4*^+^ Tfh2 cells express high *GATA-3* and *ICOS* and can produce *IL-4*, directly promoting IgG4 class switching (58). Together, these data support a dual regulatory architecture, defined as an accelerator–brake system, mediated by distinct Tfh/Tfr-related states in IgG4-RD. Disease onset and persistence may reflect an imbalance in which pro-inflammatory acceleration outweighs regulatory braking. The IgG4-RD microenvironment can thus be viewed as a counterbalanced interplay between a pro-inflammatory accelerator module (*CCR4*^+^ Tfh2, Tph, *TIGIT*^+^ Tfh and HLA^+^*GZMK*^+^ cytotoxic Tfh states) and a regulatory brake module (DP-Tfh and Tfr states). In IgG4-RD, Tfh-associated transcriptional programmes co-expand with B-cell activation and differentiation and are accompanied by upregulation of cytotoxic effector signals, suggesting that T-cell–dependent humoral responses and inflammatory effector processes jointly sustain amplification of antibody-mediated immunity and couple it to inflammatory fibrosis.

### 2.6 Rheumatoid arthritis

Rheumatoid arthritis is driven by immune-cell accumulation within the synovial microenvironment and fibroblast-subset reprogramming; here we highlight the *CXCL13*-centred T–B axis, fibroblast rewiring and bidirectional myeloid functions that together promote joint destruction and align with cross-disease shared modules. At the T-cell level, *CXCL13*^+^*CD4*^+^ T cells are increased in synovial fluid and tissue and exhibit exhaustion-like programmes consistent with chronic antigenic stimulation, forming inflammatory circuits through interactions with macrophages (59). *PDCD1*^+^ Tph and Tfh subs*ETS* express high *CXCL13* and other B-cell–recruiting and activating factors and are markedly expanded in leukocyte-rich RA (60). This suggests that in pathological subtypes with more prominent immune infiltration, the T–B axis is more likely to act as a dominant amplifier. In addition, ITGAX^+^*CD11c*^+^*TBX21*^+^ B cells—linked to T-cell recruitment—display autoimmune activation signatures and may sustain local humoral immunity by enhancing antigen presentation and co-stimulation and by participating in T-cell–dependent B-cell responses. Among fibroblasts, *FAPα*^+^ subs*ETS* show clear functional specialisation. *FAPα*^+^*THY1*^+^ fibroblasts express high levels of chemokines and pro-inflammatory cytokines, supporting immune effector functions and inflammatory driving programmes (61). *FAPα*^+^*THY1*^-^ fibroblasts upregulate *RANKL*, *CCL9*, *MMP3*, *MMP9* and *MMP13*, promoting osteoclast differentiation and activation and thereby mediating cartilage and bone erosion. Mechanistically, endothelial cells in the inflammatory milieu can activate *Notch3* signalling to reprogramme synovial fibroblasts towards pro-inflammatory and tissue-destructive states, thereby driving arthritis pathology (62). Within the myeloid compartment, synovial macrophages also partition into two major functional subs*ETS*. MerTK^+^ STM (expressing MerTK and *CD20*6) are associated with resolution, immunoregulation and fibroblast repair, whereas MerTK^-^ STM predominantly produce pro-inflammatory mediators and induce fibroblast inflammation (63). Thus, myeloid cells can shape the inflammatory and invasive state of fibroblast-like synoviocytes (FLS), contributing to synovial invasiveness and tissue destruction. *SPP1*^+^ and S100A12^+^ macrophages have also been implicated in RA by modulating inflammatory and destructive FLS phenotypes and thereby promoting joint invasion and damage (59). Notably, programmed cell death across multiple cell types contributes to RA pathogenesis, including pyroptosis, apoptosis, ferroptosis and autophagy (64, 65). These processes can promote autoantigen release and immune-complex formation, augmenting IFN-I signalling and further amplifying immune activation (66). Moreover, by strengthening myeloid–fibroblast crosstalk and propagating tissue-destructive phenotypes, programmed death pathways may act as terminal amplifiers that sustain chronicity and erosive progression. Single-cell analyses of RA synovium integrate the T–B interaction axis, fibroblast reprogramming and myeloid signalling into a unified pathogenic microenvironmental network, providing a strong theoretical basis for cross-disease target prioritisation.

## 3 Results

**Figure 2:**
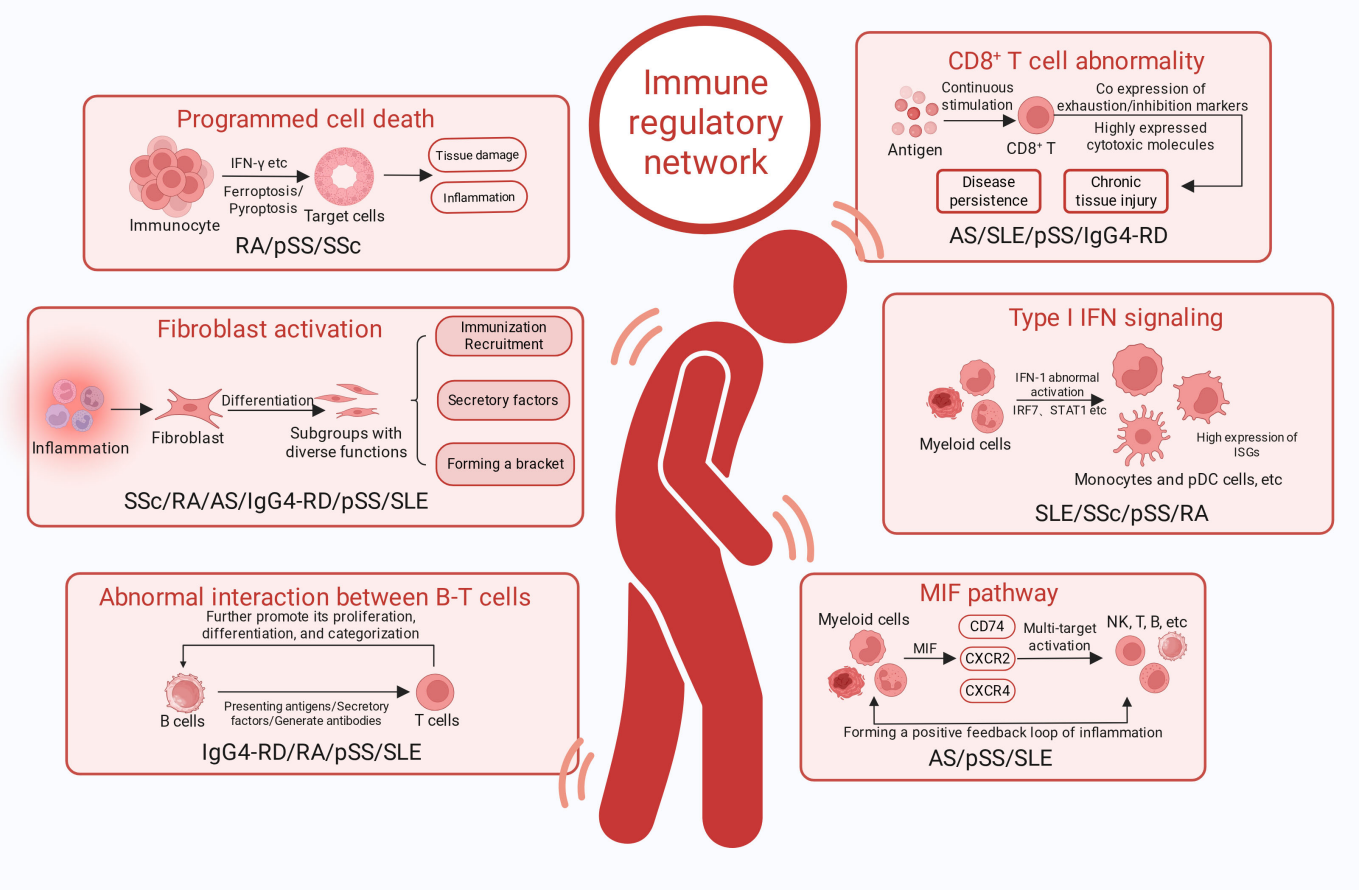
Cross disease profile of systemic sclerosis, ankylosing spondylitis, Sjogren’s syndrome, IgG4-related diseases, and systemic lupus erythematosus. Created by BioRender.

Across the six autoimmune diseases reviewed, convergent single-cell evidence suggests that these conditions can be organised into a shared pathological network across tissues and cohorts. This network can be decomposed into five recurrent modules: IFN-I signalling, aberrantly strengthened T–B interactions, a myeloid inflammatory amplification loop centred on MIF signalling, fibroblast-driven immune remodelling, and programmed cell death. Each module corresponds to reproducible cell states and communication patterns repeatedly reported across diseases and tissues. The five shared mechanisms and their correspondences are summarised in **Figure 2**. Evidence distribution across diseases and actionable targ*ETS* are summarised in **Table 1**.

**Table 1:** Cross-disease similarity at single-cell level across autoimmune diseases.

Representative cell types/states	SSc	SLE	pSS	AS	IgG4-RD	RA	Shared mechanisms	Actionable therapeutic targ*ETS*/strategies
A. IFN-I input and humoral amplification								
IFN-I/ISG-high myeloid cells (*ISG15*/*STAT1*/*IRF7*); pDC *IFIT3*–*TBK1* input; *BAFF* upregulation sustaining B-cell activation.	✓	✓	✓			✓	Upstream IFN-I input propagates via JAK–STAT; enhances antigen presentation and induces *BAFF*, sustaining a humoral amplification loop.	*IFNAR1* (anifrolumab); JAK inhibitors; *BAFF* (belimumab/telitacicept); *TLR7*/*IRF7*/*TBK1* axis (investigational)
Refs (11, 19, 24, 27, 37, 66):								
B. Cytotoxic T-cell programs								
Cytotoxic *CD8*^+^ CTL/TEMRA/Trm with *PD-1*/*TIGIT*/LAG-3 upregulation but retained effector function; *CD4*^+^ CTL (*CX3CR1*/*NKG2D*) in selected diseases.		✓	✓	✓	✓		Persistent antigenic stimulation yields cytotoxicity plus inhibitory receptors; ongoing killing/cytokines drive chronic inflammation and tissue injury.	JAK/*mTOR* pathways; checkpoint/co-stimulation modulation (*PD-1*/*TIGIT*/*OX40*; investigational/label expansion); *SLAMF7* (elotuzumab, IgG4-RD)
Refs (9, 25, 31, 36, 45):								
C. Aberrant T–B crosstalk and local humoral amplification								
*PDCD1*^+^ Tph/Tfh with *CXCL13*/*IL-21*/*ICOS*; TLS-supporting stromal cells (*CCL19*^+^ immunofibroblasts); ABC-like B cells (*CD11c*/*TBX21*).		✓	✓		✓	✓	*CXCL13* recruits B cells; *IL-21*/*ICOS* promotes differentiation; TLS enables local antigen presentation and sustained plasma-cell responses.	*CD40*/*CD40L*; *CTLA4*-Ig (abatacept); *BTK* inhibitors; *LTβR*/*NF-κB* and *BAFF*-related pathways
Refs (9, 37, 55, 59, 60):								
Representative cell types/states	SSc	SLE	pSS	AS	IgG4-RD	RA	Shared mechanisms	Actionable therapeutic targ
D. MIF-centered myeloid amplification axis								
MIF–*CD74*/*CXCR4*/*CD44* as a communication hub; in AS, T/NK-cell MIF signalling dominates the network.		✓	✓	✓			An inflammatory hub promoting recruitment and survival, forming self-amplifying loops that sustain chronic inflammation.	*CD74* targeting; *CXCR4* antagonism; MIF inhibition (antibodies/small molecules; investigational)
Refs (42, 46, 67):								
E. Fibroblast-driven immune remodelling and tissue outcome								
Activated fibroblast states (*ACKR3*^+^/*GEM*^+^/*FAPα*^+^) interact with immune cells to drive ECM deposition, erosion, or pathological ossification.	✓	✓	✓	✓	✓	✓	Fibroblasts become immune-responsive, producing chemokines/inflammatory mediators and remodelling ECM, shaping fibrotic vs destructive outcomes.	*Notch3*; *RANKL* (denosumab); *CXCL12*/*CXCR4*; *FAP* targeting (investigational)
Refs (8, 28, 48, 61, 62):								
F. Programmed cell death and damage amplification								
Ferroptosis/pyroptosis/apoptosis releasing DAMPs; coupled to IFN-I and myeloid–fibroblast crosstalk.	✓		✓			✓	DAMPs enhance antigen presentation/immune complexes, reinforcing IFN-I and further amplifying inflammation and tissue damage.	*NRF2*–xCT/*KEAP1*; inflammasome/pyroptosis inhibition; antioxidant and iron-metabolism interventions (investigational)
Refs (34, 35, 64, 68, 69):								

✓ indicates findings repeatedly reported in single-cell studies included in this review; blank indicates the module was not highlighted as a representative finding.

### 3.1 Abnormal cell metabolism

IFN-I–related pathways recur across multiple cell types and can concurrently shape innate inflammatory states and adaptive differentiation trajectories. In SSc, SLE, RA and pSS, IFN-I signalling is particularly prominent in myeloid cells, often in classical monocytes with high ISG expression (11, 19–23, 29, 30, 66, 70, 71). In RA, IFN-I exposure associates with upregulation of *TLR7*/IRF-related mediators and accompanies monocyte differentiation towards monocyte-derived dendritic cells with enhanced antigen-presentation capacity. In SSc, non-classical monocytes and *IRF7*^+^*STAT1*^+^ inflammatory monocytes also show prominent interferon responses, frequently with high *ISG15* expression. In pDCs from both SLE and pSS, *IRF7* upregulation has also been reported (12, 24, 70, 71). Across SLE, pSS, AS and IgG4-RD, cytotoxic *CD8*^+^ T cells commonly show increased effector-molecule expression alongside upregulation of exhaustion-associated receptors (9, 25, 26, 31, 32, 44, 45, 52, 58). In SLE, expansion of *CD161*^-^ TEMRA cells is observed. In pSS, *CD8*^+^ Trm cells show high *IFN-γ* and *TNFSF9* expression. In AS, T cells can upregulate inhibitory receptors while retaining cytotoxic effector function. In IgG4-RD, expansion of *GNLY*^+^ CTLs and *TIGIT*^+^*CD8*^+^ cytotoxic T cells has been reported. Cytotoxic *CD4*^+^ T cells have also been described in pSS and IgG4-RD, with potential contributions to tissue injury and fibrotic progression (36, 57, 58, 72).

### 3.2 Abnormal cell-cell interactions

Interactions between pathogenic T-cell states and B-cell differentiation trajectories are strengthened and sustained, often accompanying T-cell–dependent humoral responses and persistent plasmacytic activity. Multiple studies repeatedly implicate aberrant T–B communication in SLE, IgG4-RD, RA and pSS (9, 17, 27, 52–56, 59, 60, 67, 72). In SLE, pDC-associated *BAFF* upregulation can erode B-cell tolerance and sustain autoantibody production. In pSS and IgG4-RD, tertiary lymphoid structures provide a tissue scaffold for local B-cell activation. In pSS, *CCL19*^+^ immunofibroblasts are proposed to support TLS structure and function. In IgG4-RD, distinct Tfh/Tfr-related states modulate *IL-21*/*ICOS*-dependent B-cell differentiation and promote IgG4^+^ plasma-cell generation. In RA, a *CXCL13*-centred T–B axis dominated by *PDCD1*^+^ Tph/Tfh states recruits and sustains ABC-like B cells, establishing a self-reinforcing inflammatory circuit within synovium. Across tissue microenvironments, myeloid cells frequently construct inflammatory amplification loops through chemotaxis, stress adaptation and intercellular signalling. In AS, pSS and SLE, MIF repeatedly emerges as a communication hub, often acting via MIF–*CD74*/*CXCR4*/*CD44* to coordinate immune recruitment and sustain chronic inflammation (17, 46, 67). Collectively, these findings support MIF-centred signalling as a shared mechanism for cross-tissue inflammatory amplification.

### 3.3 Terminal fate of cells

Across SSc, SLE, pSS, AS, RA and IgG4-RD, activated fibroblasts and their immune interactions can generate tissue inflammation and bias outcomes towards fibrosis or structural damage (8, 9, 28, 37, 48, 61, 73). Each disease exhibits representative functional fibroblast states. In SSc, *ACKR3*^+^ myofibroblast precursors are linked to macrophage recruitment. In pSS, *CCL19*^+^ immunofibroblasts contribute to TLS maintenance. In SLE, *GEM*^+^ fibroblasts have been implicated in promoting tissue inflammation. In AS, FSP1^+^ fibroblasts are associated with pathological bone formation. In RA, *FAPα*^+^ fibroblast subs*ETS* drive both inflammation and erosive damage. In IgG4-RD, fibroblast activation likewise constitutes a core component of inflammatory fibrosis. Programmed cell death is prominent in pSS, SSc and RA and can interact with inflammatory signalling to amplify damage through DAMP release and antigen presentation (34, 35, 64, 68, 69). Ferroptosis and pyroptosis contribute to tissue injury in pSS and SSc. In pSS, salivary gland epithelial cells can undergo *IFN-γ*–associated ferroptosis. In SSc-ILD, lung epithelial cells may engage pyroptosis-associated pathways. In RA, multiple cell-death programmes may converge to reinforce IFN-I axes and myeloid–fibroblast interactions.

These shared mechanisms provide a mechanistic explanation for why multi-target interventions can show efficacy across distinct diseases(**Table 2**). Notably, JAK inhibitors and T-cell co-stimulation blockade have accumulated clinical evidence at different stages across multiple diseases, consistent with their capacity to modulate IFN-I–linked inflammation and T–B amplification loops (76, 80–82, 92, 93, 96–101). Therapies targeting *BAFF* (belimumab), *IFNAR1* (anifrolumab), selective JAK inhibition (e.g., upadacitinib, baricitinib), *CD40*/*CD40L* (e.g., iscalimab, dazodalibep) and B-cell depletion (rituximab) further support the premise that shared mechanistic networks can justify cross-disease repurposing and more rational combination strategies (75, 77, 78, 84, 85, 94, 102–106).

**Table 2:** Targeted drugs validated by the latest research progress in single-cell genomics, their targ*ETS* and stage.

Disease	Drug name	Drug target	Remarks
SLE	Belimumab	*BAFF*	Approved by FDA
	Telitacicept		Approved by NMPA
	Anifrolumab	*IFNAR1*	Approved by FDA
	Sirolimus	*mTOR*	Phase II trials (74)
	Upadacitinib	JAK	Phase IIb trials (75)
	Tofacitinib		Phase I trials (76)
	Baricitinib		Phase III trials (77)
SSc	Rituximab	*CD20*	Phase II trials (78) Recommended by the guide for use (79)
	Abatacept	T-cell co-stimulatory molecules	Phase IV trials (80)
	Tofacitinib	JAK	phase IIa trials (81)
SS	Abatacept	T-cell co-stimulatory molecules	Phase III trials (82)
	Iscalimab	*CD40*	Phase IIb trials (83)
	Dazodalibep	*CD40L*	Phase II trials (84)
	Baricitinib	*JAK1*/*JAK2*	Phase II trials (85)
	Filgotinib	*JAK1*	Phase II trials (86)
	Low-Dose *IL-2*	*IL-2* receptor	Phase II trials (87)
AS	Secukinumab	*IL-17*	Approved by FDA
	Ixekizumab		Approved by FDA
	Bimekizumab		Approved by FDA
	Adalimumab	*TNF*-α	Approved by FDA
	Golimumab		Approved by FDA
	Certolizumab pegol		Approved by FDA
RA	Abatacept	T-cell co-stimulatory molecules	Approved by FDA
	Dazodalibep	*CD40L*	Phase II trials (NCT07281456)
	Fenebrutinib	B cells and myeloid cells	Phase II trials (88)
	Rituximab	*CD20*	Applied to RA (89)
	Denosumab	*RANKL*	Phase III trials (90)
	Anifrolumab	*IFNAR1*	Phase IIa trials (NCT03435601)
	JAK inhibitor (Tofacitinib, Baricitinib, Upadacitinib)	JAK	Approved by FDA
Disease	Drug name	Drug target	Remarks
IgG4-RD	Dupilumab	*IL-4* receptor α	Pathological report stage (91)
	Abatacept	T-cell co-stimulatory molecules	Phase IIa trials (92)
	Tofacitinib	JAK-STAT	Pathological report stage (93)
	Upadacitinib		Pathological report stage (94)
	Elotuzumab	*SLAMF7*	Phase II trials (NCT04918147)
	Rituximab	*CD20*	Phase II trials (NCT01584388) Recommended by the guide for use (95)

## 4 Discussion

A central premise of this review is that, at single-cell resolution, autoimmune diseases with distinct phenotypes and target organs can nevertheless share core pathogenic mechanisms. The six diseases discussed can be conceptualised as an integrated network comprising upstream inflammatory inputs, immune-cell amplification loops and tissue-level pathological remodelling. Within this network, IFN-I inputs, T–B crosstalk, MIF-centred myeloid signalling, fibroblast immune responsiveness and programmed cell death act in concert to sustain chronic inflammation and drive organ-specific injury (**Figure 2**). Shared mechanisms provide a theoretical basis for the efficacy of pathway-convergent drugs across diseases and for other mechanism-informed cross-disease strategies. The recurrence of IFN-I signatures in myeloid and dendritic compartments suggests a relatively upstream position, capable of lowering activation thresholds, establishing inflammatory tone and influencing antigen presentation early in disease. On this background, sustained T–B interactions not only maintain antibody-secreting responses but also propagate inflammation through diverse cytokine circuits across blood and tissue. Concurrently, myeloid cells coordinate recruitment, maintain inflammatory states and stabilise local microenvironments. Fibroblast immune responsiveness mediates tissue pathology and drives immune-architectural remodelling. Programmed cell death releases damage-associated signals that close amplification loops linking inflammation to tissue destruction. Distilling these observations, we prioritise cross-disease targ*ETS* repeatedly identified at single-cell resolution, largely converging on a small set of hub pathways: the MIF–*CD74*/*CXCR4*/*CD44* inflammatory amplification axis; the *CXCL13*–*PDCD1*^+^ Tph/Tfh–*IL-21*/*ICOS* T–B amplification axis; and the IFN-I (*TLR7*/*IRF7*/*STAT1*)–*BAFF* humoral axis. Co-stimulatory regulators such as *TIGIT* and *OX40* may further serve as combinatorial intervention nodes.

From a translational perspective, these pathways sit at key communication bottlenecks and therefore offer favourable druggability. They include antibody-accessible surface molecules (e.g., *CD74*, *TIGIT*, *OX40* and *BAFF*) as well as small-molecule–tractable innate sensing pathways (e.g., *TLR7*/8-related signalling). Notably, several targ*ETS* already have clinical or near-clinical validation in oncology or fibrotic disease. For example, anti-*CD74* antibodies have been explored in haematological malignancies; *TIGIT* and *OX40* modulators have entered solid-tumour immunotherapy trials; and *CXCR4* antagonism has been tested to remodel immune microenvironments in diseases such as pancreatic cancer (107–111). In parallel, non-response to existing targeted therapies highlights that single-node intervention may be insufficient across heterogeneous tissue contexts (112). Accordingly, longitudinal single-cell and spatial multi-omics that link pathways, cell states and clinical outcomes will be required for patient stratification, response prediction and identification of more upstream, broadly applicable intervention points to support cross-disease therapy.

Within this integrative network, cross-disease target prioritisation can follow three principles. First, whether the target is consistently activated across diseases, tissues and cell types. Secondly, consider the position of the target in the mechanism. Third, the strength of association with clinical phenotype, disease activity or treatment response. Targ*ETS* that are cross-disease stable and operate upstream are better suited for cross-disease trials. By contrast, downstream nodes—such as specific fibroblast subs*ETS* or cell-death programmes—may be better positioned as organ-contextual intervention points. This framework also supports rational combinations that pair upstream inflammation control with downstream tissue-protection strategies. Single-cell evidence offers direct mechanistic explanations for multi-disease efficacy of certain agents (**Table 2**), including JAK inhibitors and abatacept, and highlights translational potential for targ*ETS* such as *BAFF*, *IFNAR1* and *CD40*/*CD40L*. Importantly, these data can inform future trial optimisation, not only by explaining past outcomes but also by shaping enrolment and endpoint strategies. Stratifying enrolment by dominant cellular programmes—myeloid IFN-I signatures, strong T–B crosstalk, fibroblast remodelling predominance—may improve response rates and clarify mechanisms of action. Longitudinal profiling before and after therapy may further reveal whether benefit reflects network-level rewiring (e.g., attenuation of T–B loops or decline of myeloid hubs) rather than changes in isolated markers.

Although single-cell technologies have enabled major discoveries, current evidence remains constrained by multiple limitations. Technical constraints include cost, batch effects, low nucleic-acid content per cell, and complex data integration; spatial omics additionally faces trade-offs between single-cell resolution and RNA capture efficiency, as well as challenges in segmentation and matrix construction (113–115). These factors can bias detection of rare states and limit cross-study comparability. Biological heterogeneity and study design are equally important sources of variation. Differences in sampled tissues and disease stages, along with treatment exposure and comorbidities, can introduce confounding. Moreover, largely cross-sectional sampling constrains stronger inference about network hierarchy and causality.

In summary, based on single-cell omics evidence, we propose that although SSc, SLE, pSS, AS, IgG4-RD, and RA differ in clinical phenotypes and target organs, they share convergent pathogenic programs encompassing IFN-I signalling, aberrant T–B cell crosstalk, amplification of myeloid-driven inflammation, fibroblast-mediated immune remodelling, and dysregulated programmed cell death, thereby providing a mechanistic rationale for cross-disease therapeutic strategies. However, current clinical therapies still fail to achieve optimal efficacy in a substantial proportion of patients, underscoring the need for continued interrogation of disease-initiating and disease-propagating mechanisms to identify more actionable therapeutic targets. We anticipate that, with ongoing advances in relevant technologies and analytical frameworks, these mechanistic insights will be translated more efficiently into clinical applications, ultimately improving therapeutic outcomes across autoimmune diseases.

## Data Availability

The original contributions presented in the study are included in the article/supplementary material. Further inquiries can be directed to the corresponding author.

## Glossary

AS: ankylosing spondylitis
*BAFF*: B cell–activating factor (*TNFSF13B*)
*BTK*: Bruton tyrosine kinase
CD: cluster of differentiation
CTL: cytotoxic T lymphocyte
*CXCL13*: C-X-C motif chemokine ligand 13
*CXCR4*: C-X-C motif chemokine receptor 4
DAMPs: damage-associated molecular patterns
DCs: dendritic cells
EndoMT: endothelial-to-mesenchymal transition
ECM: extracellular matrix
*FAPα*: fibroblast activation protein alpha
FLS: fibroblast-like synoviocytes (synovial fibroblasts)
IFN-I: type I interferon
IFN-α: interferon alpha
*IFN-γ*: interferon gamma
*IFNAR1*: type I interferon receptor subunit 1
IgG4-RD: IgG4-related disease
IL: interleukin
ISG(s): interferon-stimulated gene(s)
JAK: Janus kinase
JAK–STAT: Janus kinase–STAT signalling pathway
*LTβR*: lymphotoxin beta receptor
MIF: macrophage migration inhibitory factor
moDC: monocyte-derived dendritic cell
*mTOR*: mechanistic target of rapamycin
*NF-κB*: nuclear factor kappa B
pDCs: plasmacytoid dendritic cells
PBMCs: peripheral blood mononuclear cells
pSS: primary Sjögren syndrome
RA: rheumatoid arthritis
*RANKL*: receptor activator of *NF-κB* ligand
SLE: systemic lupus erythematosus
SS: Sjögren syndrome
SSc: systemic sclerosis
SSc-ILD: systemic sclerosis–associated interstitial lung disease
STM: synovial tissue macrophages
TEMRA: terminally differentiated effector memory T cells re-expressing *CD45RA;* Tfh, T follicular helper cells
Tfr: T follicular regulatory cells
*TIGIT*: T cell immunoreceptor with Ig and ITIM domains
TLO: tertiary lymphoid organ
TLS: tertiary lymphoid structure
Tph: peripheral helper T cells
Trm: tissue-resident memory T cells
scRNA-seq: single-cell RNA sequencing
scATAC-seq: single-cell ATAC sequencing.
